# Electroneurological changes in peripheral nerves in patients post-COVID

**DOI:** 10.1152/jn.00396.2022

**Published:** 2022-12-14

**Authors:** Jakub Stępień, Żanna Pastuszak

**Affiliations:** ^1^Insula Clinical Trials Center, Warsaw, Poland; ^2^Laboratory of Experimental and Clinical Neurosurgery, Mossakowski Medical Research Institute Polish Academy of Sciences, Warsaw, Poland; ^3^Department of Neurosurgery, Bielanski Hospital, Warsaw, Poland

**Keywords:** coronavirus, electroneurography, nerve conduction study, neuropathy, post-COVID-19 neuropathy

## Abstract

Various neurological manifestations are observed in about 36.4% of patients infected with SARS-CoV-2 and post-COVID neuropathy is one of them. There is lack of studies describing neurophysiological abnormalities in peripheral nerves in case of patients who had SARS-CoV-2 infection. The aim of this study was to analyze the changes in peripheral nervous system in case of COVID-19 survivors. In the presented study, 45 COVID-19 survivors who had nerve conduction study (NCS) were involved. Results were compared with control group consisting of healthy patients who had nerve conduction study before the COVID-19 pandemic. In our study group, neurophysiological abnormalities were present in the case of both sensory and motor nerve fibers. The most significant reduction of NCS parameters was observed in the case of sensory action potential amplitude of sural nerve. Moreover, that correlation was the most significant in the case of amplitude and conduction velocity in sensory and motor neuron fibers both in arms and legs. Those abnormalities were observed even 6 mo after COVID-19. Further investigation needs to be done regarding the polyneuropathies associated with human coronaviruses, and we should answer the question whether the virus directly damages peripheral nerves or factors mediating inflammatory response are responsible for the neural damage.

**NEW & NOTEWORTHY** Various neurological manifestations are observed in about 36.4% of patients infected with SARS-CoV-2 and post-COVID neuropathy is one of them. There is lack of studies describing neurophysiological abnormalities in peripheral nerves in case of patients who had SARS-CoV-2 infection. The aim of this study was to analyze changes in peripheral nervous system in case of COVID-19 survivors.

## Graphical abstract

**Figure:**
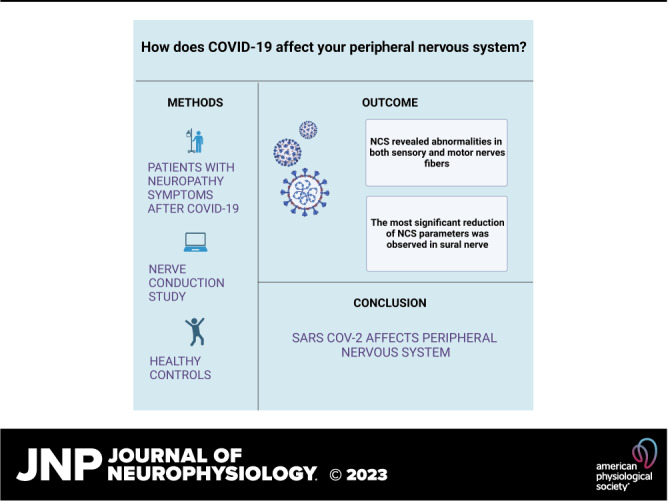

## INTRODUCTION

The predominant clinical presentation COVID-19 infection is a respiratory disease with the most common complications including pneumonia and acute respiratory distress syndrome. Few studies have shown that also central nervous system (CNS) and peripheral nervous system disease caused by SARS-CoV-2 might be more frequent than we thought before. In another study, the most common neurological symptoms reported in patients with COVID-19 were headache (6%), vertigo (3.4%), paresthesia (3.1%), altered consciousness (2%), hyposmia/anosmia (1.4%), and encephalitis (0.9%), cerebrovascular events (0.6%), seizure (0.3%), Guillain–Barré syndrome (GBS) (0.3%), and several case reports of Miller Fisher syndrome and other neuropathies (1). Rhabdomyolysis, myopathy, myositis, myasthenia, myasthenic syndrome as well as polyradiculitis may also be connected to SARS-CoV-2 infection but their incidence remains unknown (2). Although various neurological manifestations are observed in ∼36.4% of patients infected with SARS-Cov-2, the incidence of post-COVID neuropathy remains unknown (3). In another study, peripheral neuropathy was observed in less than 1% of patients with COVID-19 (4). Risk factors for neuropathy identified were diabetes, obesity, drug use, and prolonged stay in the intensive care unit (2). One of the most important goal of that study was to differentiate patients with critical illness polyneuropathy and myopathy (CIAW) from post-COVID neuropathy. CIAW is a neuromuscular disorder that may develop in patients with severe disease often resulting in admission to an intensive care unit. COVID-19 infection is a risk factor of CIAW and in case of patients with severe course of COVID-19 it may be difficult to differentiate post-COVID neuropathy from CIAW (5).

Etiopathogenesis of peripheral neuropathy in patients with post-COVID is unknown. Molecular mimicry, which is an important mechanism in creating autoimmune disorders, may have a role in the development of COVID-19-associated polyneuropathy. Moreover, in COVID-19 hyperinflammation and immune system dysregulation are observed and can be also responsible for peripheral nerves damage. Some evidence suggests that hyperinflammation in case of patients with COVID-19 is associated with increased interleukin (IL)-2, IL-7, granulocyte-colony stimulating factor, interferon-γ inducible protein 10, monocyte chemoattractant protein 1, macrophage inflammatory protein 1-α, and tumor necrosis factor-α. All mentioned cytokines are connected to severe disease course and are predictors of fatality rate (6–8).

## CLINICAL RATIONALE FOR THE STUDY

There is a lack of studies describing neurological complications in COVID-19 survivors that were made on bigger groups of patients with the use of nerve conduction study (NCS). Moreover, there is a need to explore the abnormalities in nerve conduction study (NCS) in patients with post-COVID-19 neuropathies. The aim of this study was to analyze changes in peripheral nervous system in case of COVID-19 survivors with neuropathy symptoms.

## MATERIALS AND METHODS

The study group consisted of 45 patients (M20, W25). The mean age in study group was 54.4 yr. The control group consisted of 45 patients (M11, W34). The mean age in the control group was 52.6 yr (Table 1).

**Table 1.** A comparison of the ages of the study group with the control group

	Group	*n*	M	SD	Med	Min.	Max.
Age	Study	45	54.4	11.4	55	22	69
	Control	45	52.6	21.8	54	23	87

M, mean age; Med, median age, Min., minimum age; Max, maximum age; *n*, number of patients; SD, standard deviation.

The study group patients who survived COVID-19 in maximal time of 6 mo before NCS study were included. All those patients were referred to our center for evaluation of neurological symptoms of post-COVID infection. All investigated patients reported lower limbs paresthesia and 17 patients had upper limb paresthesia. Sensory peripheral nerve symptoms were present in case of 11 patients and muscle weakness that started after COVID-19 infection was present in case of three patients. COVID-19 infection was confirmed with the use of PCR test. The study group patients with mild to moderate SARS-Cov-2 infection were included. All patients had respiratory tract infection with the symptoms like fever, cough, sore throat, general weakness, and muscle pain. Patients with severe disease course who were hospitalized in intensive care unit were excluded from the study. In this group of people, peripheral nerve damage could be connected to the presence of CIAW or to prone position during respiratory therapy. All patients fulfilled the criteria of the long-COVID according to NICE (9, 10). Patients treated with neurotoxic drugs such as daptomycin, linezolid, lopinavir, ritonavir, hydro-chloroquine, cisatracurium, clindamycine, and glucocorticoids were also excluded from the study due to the possibility of inducing toxic neuropathy. Patients treated with neurotoxic drugs as well as patients with other diseases that could be connected to polyneuropathy such as diabetes, vitamin B12 deficiency, or alcohol abuse were excluded from the study. Patients were asked by the doctor about their medical history and actual symptoms to exclude diseases that could cause polyneuropathy. Patients were kept under medical supervision by GP doctors and had periodical laboratory tests. Patients were excluded from the study in case of abnormal findings on examination of blood that could be connected to polyneuropathy.

Patients in the study group did not have any signs and symptoms of peripheral nerve damage before SARS-CoV 2 infection.

Control group consisted of healthy volunteers who did not have symptoms of any neurologic disorder and abnormalities during neurological examination. Patients had nerve conduction study since 2014 to 2018 when SARS-COV-2 infection wasn’t present in the population. Those people participated in study where nerve conduction study was evaluated in patients with multiple sclerosis and results were compared with control group consisted of healthy volunteers. Patients in control group were age-matched and sex-matched for patients in the investigated group. Patients with any abnormalities in the neurological examination as well as patients diagnosed with any disorder or taking any drugs were excluded from the study.

Bioethics committee and regulatory approval were obtained before the study was started and in case of all patients the written informed consent was taken.

All patients in the investigated and control group had NCS. Both sensory and motor fibers of right median, ulnar, fibular as well as sural nerve were investigated with the use of NCS. Motor and sensory nerve conduction study was performed in an environment with temperature ranging between 21 and 25°C. Amplitude, conduction velocity as well as distal latency and F-wave latency were examined and compared with results in control group with the use of Mann–Whitney test (11). Sensory studies were performed antidromically. In the case of median nerve sensory action potential, the recording electrode was placed on the second finger while in case of ulnar nerve it was placed on the fifth finger. The sural nerve was stimulated on the posterolateral aspect of the lower third of the leg and the distance between the recording electrode and the stimulating one was ∼14 cm. Sensory nerve action potential amplitudes were measured baseline-to-peak. In case of median nerve motor fibers, the recording electrode was placed over the abductor pollicis brevis muscle whereas in the case of ulnar nerve it was placed over the adductor digiti minimi muscle. In case of fibular nerve, the recording electrode was placed over the extensor digitorum brevis whereas the abductor hallucis muscle was the placement of recording electrode in case of tibial nerve. The distance between the recording electrode and place of stimulation was 8 cm. Locations of stimulating and recording electrodes in case of all examined nerves were typical as presented in those studies (12, 13).

## RESULTS

### Median Nerve

**Figure 1.**
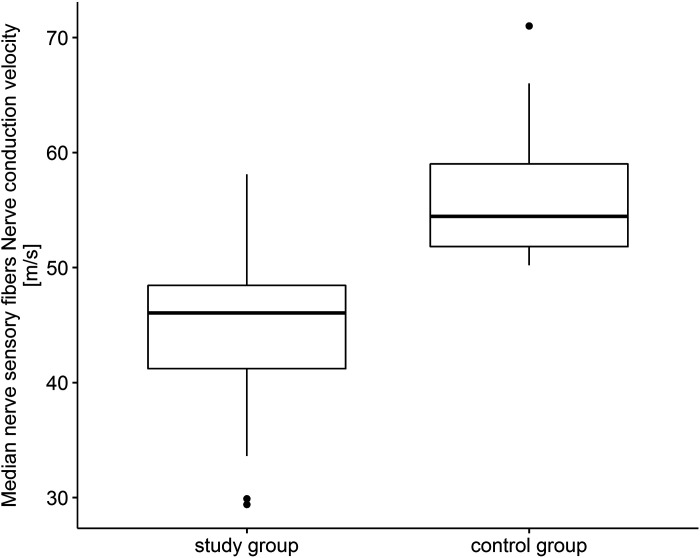
Median nerve sensory fibers. Nerve conduction velocity (m/s).

The median sensory nerve action potential amplitude in patients with COVID-19 was lower compared with the control group (*U* = 332.5, *P* < 0.001). Conduction velocity in sensory fibers of median nerve was also lower compared with control group (*U* = 75.5, *P* < 0.001) (Fig. 1). In case of median nerve motor fibers, in study group action potential amplitude was statistically lower than that in the control group as well as the conduction velocity (*U* = 659.5, *P* = 0.007). The same situation was in the case of distal latency (*U* = 321.5, *P* < 0.001) as well as F-wave latency that were statistically higher compared with control group (*U* = 575.0, *P* < 0.001). The strongest correlation was in the case of nerve conduction velocity reduction in the study group compared with other nerve conduction study parameters (amplitude, distal latency, and F-wave latency) (*r* = 0.93).

### Ulnar Nerve

**Figure 2.**
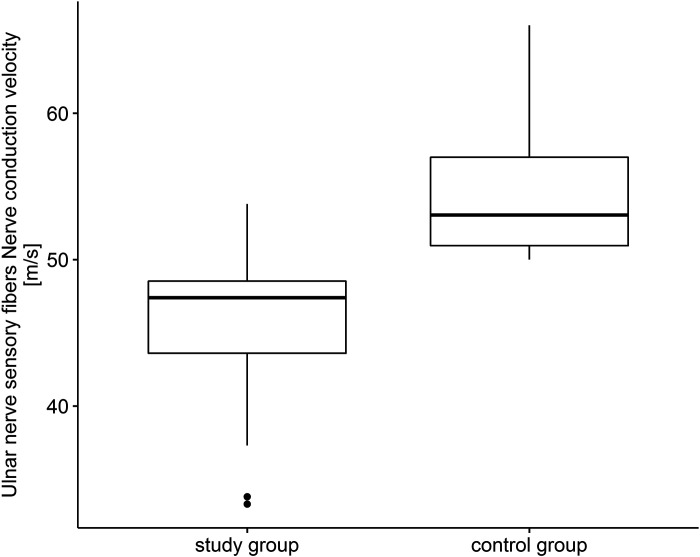
Ulnar nerve sensory fibers. Nerve conduction velocity (m/s).

Statistical analysis revealed that sensory nerve action potential amplitude of ulnar nerve in patients with COVID-19 was lower compared with controls (*U* = 383.0, *P* < 0.001). Conduction velocity in sensory fibers of ulnar nerve was also lower compared with control group (*U* = 80.5, *P* < 0.001) (Fig. 2). In case of motor fibers of ulnar nerve in the study group, action potential amplitude was statistically lower than that in the control group (*U* = 621.5, *P* = 0.009) as well as the conduction velocity (*U* = 263.5, *P* < 0.001). The same situation was in the case of distal latency (*U* = 572.0, *P* = 0.002) as well as F-wave latency that were statistically higher compared with control group (*U* = 642.0, *P* = 0.015). The strongest correlation was in the case of nerve conduction velocity reduction in the study group compared with controls (*r* = 0.91).

### Fibular Nerve

**Figure 3.**
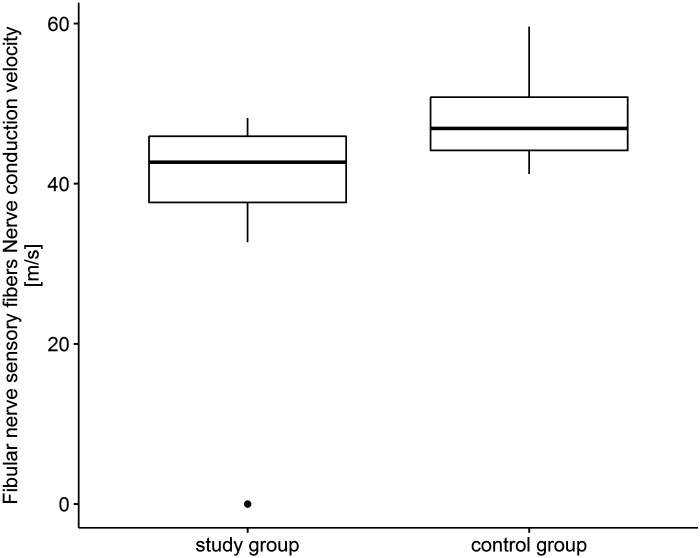
Fibular nerve sensory fibers. Nerve conduction velocity (m/s).

The fibular sensory nerve action potential amplitude in patients with COVID-19 was lower compared with controls (*U* = 38.0, *P* < 0.001). Conduction velocity in sensory fibers of fibular nerve was lower compared with control group (*U* = 353.5, *P* < 0.001) (Fig. 3). In case of motor fibers of fibular nerve in the study group, action potential amplitude was statistically lower than that in the control group (*U* = 440.5, *P* < 0.001) as well as the conduction velocity (*U* = 271.0, *P* < 0.001). The same situation was in case of distal latency (*U* = 730.5, *P* < 0.023) as well as F-wave latency that were statistically higher compared with control group (*U* = 641.0, *P* = 0.003). The strongest correlation was in the case of sensory action potential amplitude reduction in the study group compared with controls (*r* = 0.96).

### Sural Nerve

**Figure 4.**
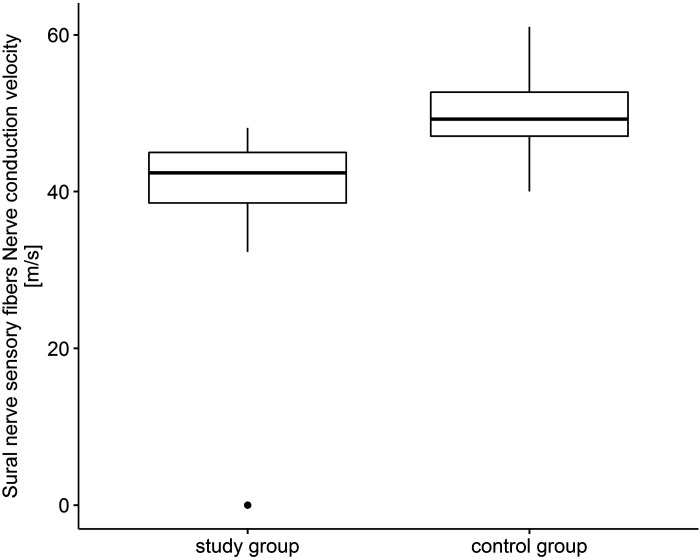
Sural nerve sensory fibers. Nerve conduction velocity (m/s).

Statistical analysis revealed that sensory nerve action potential amplitude in patients with COVID-19 was lower compared with controls (*U* = 0.0, *P* < 0.01). The same situation was in the case of conduction velocity that was lower compared with the control group (*U* = 172.0, *P* < 0.01) (Fig. 4). The strongest correlation was in the case of sensory action potential amplitude reduction in the study group compared with controls (*r* = 1.0).

### Tibial Nerve

The motor nerve action potential amplitude of tibial nerve was lower in patients with COVID-19 compared with controls (*U* = 532.5, *P* < 0.001). Conduction velocity in motor fibers of tibial nerve was also lower compared with control group (*U* = 353.5, *P* < 0.001). In the case of motor fibers of tibial nerve in the study group, action potential amplitude was statistically lower than that in the control group (*U* = 440.5, *P* < 0.001) as well as conduction velocity (*U* = 209.5, *P* < 0.001). The same situation was in the case of F-wave latency that was statistically higher than that in the control group (*U* = 577.0, *P* = 0.001). The strongest correlation was in the case of nerve conduction velocity reduction in the study group compared with controls (*r* = 0.79). The values of NCS parameters in study and control group are presented in Tables 2 and 3.

**Table 2.** Basic descriptive statistics for the nerves of the upper extremities

	Group	*n*	M	SD	Me	IQR	*P* Value
**Median nerve sensory fibers response amplitude, µV**	Study group	45	12.37	8.204			<0.001
	Control group	45	30.09	18.235			
**Median nerve sensory fibers nerve conduction velocity, ms**	Study group	45	44.62	6.213			<0.001
	Control group	45	56.03	5.011			
Median nerve motor fibers distal latency (between APB and wrist), ms	Study group	45			3.87	0.750	<0.001
	Control group	44			3.43	0.428	
Median nerve motor fibers response amplitude (between APB and wrist), mV	Study group	45			6.80	3.800	0.007
	Control group	44			8.25	3.900	
**Median nerve motor fibers nerve conduction velocity (between APB and wrist), ms**	Study group	45	53.75	3.936			<0.001
	Control group	44	59.13	4.141			
**Median nerve motor fibers F wave latency, ms**	Study group	45	27.77	2.401			<0.001
	Control group	44	25.92	2.202			
Ulnar nerve sensory fibers response amplitude, µV	Study group	45			12.90	6.500	<0.001
	Control group	41			25.00	28.400	
**Ulnar nerve sensory fibers nerve conduction velocity, ms**	Study group	45	46.07	4.440			<0.001
	Control group	41	54.58	4.187			
Ulnar nerve motor fibers distal latency (between ADM and wrist), ms	Study group	45			3.15	0.470	0.002
	Control group	41			2.80	0.370	
Ulnar nerve motor fibers response amplitude (between ADM and wrist), mV	Study group	45			8.90	2.600	0.009
	Control group	41			10.20	2.400	
Ulnar nerve motor fibers nerve conduction velocity (between ADM and wrist), ms	Study group	45			54.20	3.300	<0.001
	Control group	41			60.00	8.000	
**Ulnar nerve motor fibers F wave latency, ms**	Study group	45	28.07	2.752			0.015
	Control group	41	26.32	2.410			

Bold text—normal distribution (rest—nonnormal distribution).

APB, abductor pollicis brevis; ADM, adductor digiti minimi; IQR, interquartile range; M, mean; Me, median; *n*, number of valid observations; SD, standard deviation.

**Table 3.** Basic descriptive statistics for the nerves of the lower extremities

	Group	*n*	M	SD	Me	IQR	*P* Value
Fibular nerve sensory fibers response amplitude, µV	Study group	45			4.60	2.000	<0.001
	Control group	45			8.20	3.700	
Fibular nerve sensory fibers nerve conduction velocity, m/s	Study group	45			42.50	8.100	<0.001
	Control group	45			48.00	7.100	
Fibular nerve motor fibers distal latency (between EDB and ankle), ms	Study group	45			4.29	0.700	0.023
	Control group	45			3.83	0.840	
**Fibular nerve motor fibers action potential amplitude (between EDB and ankle), mV**	Study group	45	**3.37**	**2.139**			<0.001
	Control group	45	**5.54**	**2.151**			
Fibular nerve motor fibers nerve conduction velocity (between EDB and ankle), ms	Study group	45			41.90	6.200	<0.001
	Control group	45			48.00	6.000	
Fibular nerve motor fibers F wave latency, ms	Study group	45			50.50	5.700	0.003
	Control group	45			47.00	5.300	
Sural nerve sensory fibers response amplitude, µV	Study group	44			4.95	2.250	<0.001
	Control group	44			12.15	7.125	
Sural nerve sensory fibers nerve conduction velocity, ms	Study group	44			42.40	6.450	<0.001
	Control group	43			49.50	7.250	
Tibial nerve motor fibers distal latency (between AHM and ankle joint), ms	Study group	44			3.61	1.047	0,634
	Control group	45			3.61	0.930	
Tibial nerve motor fibers action potential amplitude (between AHM and ankle joint), mV	Study group	44			10.00	7.000	<0.001
	Control group	45			13	7.000	
Tibial nerve motor fibers nerve conduction velocity (between AHM and ankle joint), ms	Study group	44			41.00	5.000	<0.001
	Control group	45			47	4.000	
Tibial nerve motor fibers F wave latency, ms	Study group	44			51.00	6.000	<0.001
	Control group	45			47	5.000	

Bold text—normal distribution (rest—nonnormal distribution).

AHM, abductor hallucis muscle; EDB, extensor digitorum brevis; IQR, interquartile range; M, mean; Me, median; *n*, number of valid observations; SD, standard deviation.

In the study group in neurological examination, decreased deep tendon reflexes were the most frequent abnormality. Sensory loss or limb weakness was more rare.

**Figure 5.**
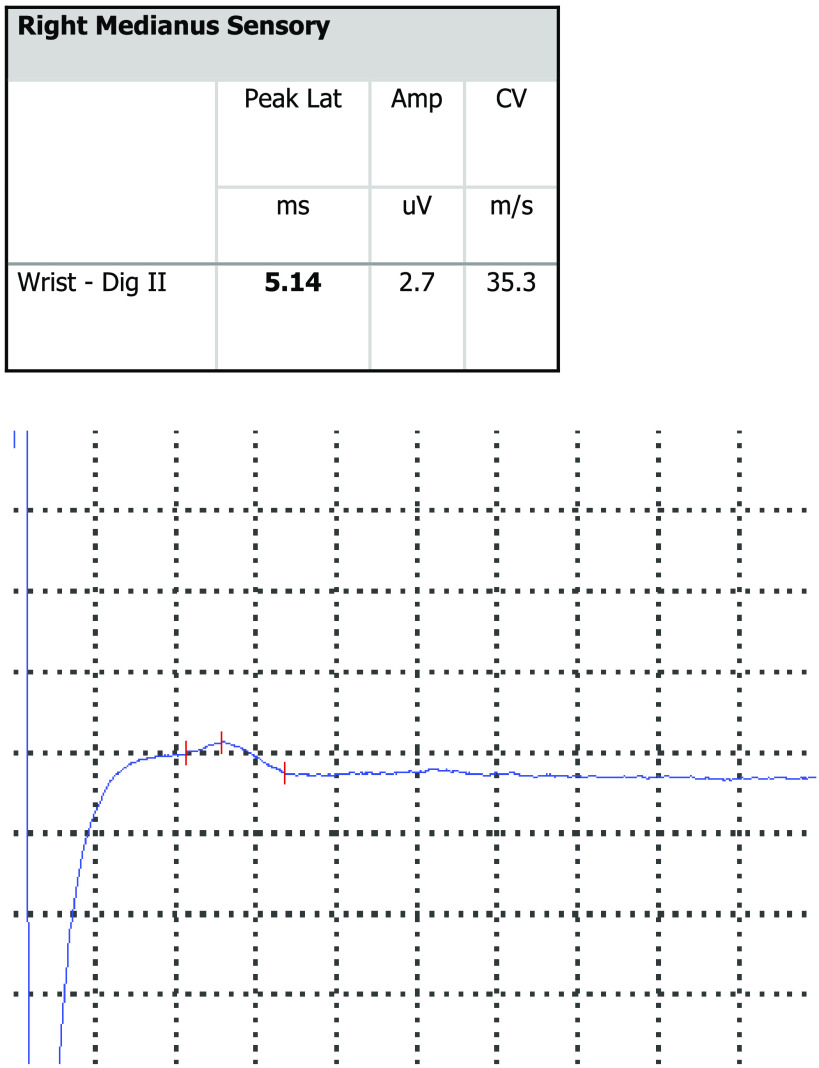
Sample nerve conduction study (NCS) result—right medianus. Amp, amplitude; CV, conduction velocity; Dig II, digit II; Peak Lat, peak latency.

Sample NCS result is presented in Fig. 5.

## DISCUSSION

In the presented study, we analyzed nerve conduction study of patients who had polyneuropathy symptoms that started during COVID infection or to 6 mo after COVID-19 infection (in case of majority of them symptoms stared during first few weeks since COVID-19 diagnosis was made). In our study, there was statistically significant reduction of amplitude and conduction velocity in both sensory and motor neuron fibers compared with control group in all examined nerves in upper and lower limbs. Moreover, distal latency and F wave latency was also increased compared with control group. All this abnormalities are typically for polyneuropathy and our results are similar to those described in the literature. The majority of published cases thus far of peripheral nervous disease associated with COVID-19 have shown a symmetrical pattern and it was mixed sensorimotor neuropathy (14–18). In our study group, neurophysiological abnormalities were present in case of both sensory and motor nerves fibers. The most significant reduction of NCS parameters was observed in case of sensory action potential amplitude of sural nerve. Moreover, that correlation was the most significant in case of amplitude and conduction velocity in sensory and motor neuron fibers both in arms and legs. In the study group, NCS showed both axonal loss and demyelinating features in COVID-19 survivors who had the symptoms of polyneuropathy. Primary demyelination can be defined as a process in which there is the destruction of myelin sheaths whereas axons are relatively spared. Secondary demyelination occurs as a consequence of axonal destruction, where the initial event is axonal damage, and myelin degeneration follows degeneration of the axons (19). In the presented study, electroneurographical parameters have indicated that secondary rather than primary demyelination is connected to post-COVID neuropathy in the study group. In another study it was revealed that among evaluated patients with symptoms of polyneuropathy after COVID-19, the small-fiber neuropathy was the most common type of peripheral nervous system disease. Symptoms were beginning within 1 mo of COVID-19 onset and diagnosis was confirmed with the use of skin biopsy (20). In our study it was obvious that SARS-Cov-2 can impair peripheral nerves fibers resulting in polyneuropathy. Etiopathogenesis of peripheral neuropathy in patients with post-COVID is unknown. Molecular mimicry as well as hyperinflammation and immune system dysregulation can be responsible for peripheral nerves damage in COVID-19 survivors. Some evidence suggests that interleukin (IL)-2, IL-7, granulocyte-colony stimulating factor, interferon-γ, inducible protein 10, monocyte chemoattractant protein 1, macrophage inflammatory protein 1-α, and tumor necrosis factor-α are the most important cytokines connected to peripheral nerves damage (6–8). The exact mechanism of entry into the central nervous system (CNS) is also unknown. Although SARS-Cov-2 may have direct access to the CNS, only two cases have been reported with SARS-Cov-2 in cerebrospinal fluid (1, 2). Currently discussed routes include retrograde neuronal transport across infected neurons, entry via the olfactory nerve, infection of the vascular endothelium, or white blood cell migration across the blood-brain barrier (21–23). Further medical investigation should be done to answer the question if those mechanism present in CNS are connected to peripheral nerves damage. Although the exact mechanism of inducing polyneuropathy in patients with post-COVID remains undiscovered, when evaluating patients with new-onset peripheral neuropathy, screening for recent COVID-19 infection should be done.

It remains unclear which factors are associated with increased risk of neurologic manifestation in case of patients with post-COVID. In another study it was suggested that neurological symptoms are more common in patients with severe disease course (3). Patients with COVID-19 are in risk group of developing CIAW. In case of patients with severe course of COVID-19 who are admitted to intensive care unit, post-COVID neuropathy can coexist with CIAW. Due to that we excluded from the study patients that fulfil the criteria of CIAW (5). Patients treated with neurotoxic drugs such as daptomycin, linezolid, lopinavir, ritonavir, hydro-chloroquine, cisatracurium, clindamycine, and glucocorticoids were also excluded from the study due to the possibility of inducing toxic neuropathy. Patients treated with neurotoxic drugs as well as patients with other disease that could be connected to polyneuropathy like diabetes, vitamin B12 deficiency, or alcohol abuse were excluded from the study. Patients in study group did not have any sign and symptoms of peripheral nerves damage before SARS-CoV 2 infection. Although in our study we have tried to rule out patients with other causes of polyneuropathy, there is need to find clear direct pathophysiological link between COVID-19 infection and neuropathy (e.g., supportive nerve biopsy findings).

In another study it was discovered that zinc deficiency can lead to cytokine production and can increase the risk of polyneuropathy in SARS-CoV-2 infection. It may be a cause of hyperinflammation present in patients with severe disease course (24). In another study it was revealed that a significant number of patients with COVID-19 were zinc deficient and the deficiency was associated with a prolonged hospital stay and increased mortality. Zinc can regulate leukocyte immune response by alerting cytokine production and has a positive role in inflammatory conditions (25). Possible connection between zinc deficiency and a possible neurotropism of SARS-CoV-2, and the mechanism of COVID-19 related neuropathy need to be fully evaluated.

## FUTURE DIRECTIONS

Further investigation, including needle EMG, needs to be done regarding the polyneuropathies associated with human coronaviruses and regarding whether the virus directly damages peripheral nerves or factors mediating inflammatory response are responsible for the neural damage. Awareness of this complication will have an impact on the treatment of patients with neurological complications of SARS-CoV-2.

## DATA AVAILABILITY

Data will be made available upon reasonable request.

## DISCLOSURES

No conflicts of interest, financial or otherwise, are declared by the authors.

## AUTHOR CONTRIBUTIONS

J.S. and Ż.P. conceived and designed research; performed experiments; analyzed data; interpreted results of experiments; prepared figures; drafted manuscript; edited and revised manuscript; approved final version of manuscript.
